# Social cohesion in psychiatry training in the UK More than a number game? – a secondary review

**DOI:** 10.1192/j.eurpsy.2023.1006

**Published:** 2023-07-19

**Authors:** V. Sathyanarayanan, M. W. Azeem

**Affiliations:** 1 University College Cork, Cork, Ireland; 2 Sidra Medical Hospital, Doha, Qatar

## Abstract

**Introduction:**

With the growing mental health concerns in the United Kingdom, it is undisputable that there will be a need for more mental health professionals, particularly psychiatrists. It has been estimated that there is 1 consultant psychiatrist per 12,567 people in England. The Royal College of Psychiatrists continue to call for a long term plan to address the mental health crisis by investing in psychiatric training and education.

**Objectives:**

To analyze the socio-demographic profile of doctors in training in psychiatry and change in demand over the last decade (2012-2021)

**Methods:**

We carried out secondary review of the state of medical education and practice in the UK in 2021 report. We analyzed reference tables in the report pertaining to doctors in training in psychiatry by age group; gender; by ethnicity; and by place of primary medical qualification. The report included data for the period 2012-13 to 2020-21, which was analyzed. In addition, 5-year (2017-2021) and 10-year (2012-2021) trends were also available and included in our analysis.

**Results:**

While in 2012, there were 1,370 doctors in training in psychiatry, the number had dropped marginally to 1,352 in 2021. While there has been an overall decline of 1.3% in the decade (2012-21), there has been a 11.1% increase (2017-21) in the last 5 years. A big change was noticed with respect to gender. While there were 670 male doctors in training in psychiatry in 2012, the number dwindled to 522 in 2021 (22.1% decline). A deeper analysis of the decline in the male psychiatry trainees reveals that the steepest decline has happened among male international medical graduates (389 to 131 or 58.1% to 25.1%). However, the 5-year trend for male doctors has been more favorable with a 10.6% increase between 2017 and 2021. Among female doctors on the other hand, there was a 18.6% increase from a baseline of 700 in 2012 to 830 in 2021. From an ethnicity perspective, there has been a sharp decline in the proportion of Asian or Asian British trainees, down by 33.9% (555 to 367) between 2012 and 21, compensated largely by White trainees, where a 50.1% increase (511 to 767) has been seen. There has been a significant fall in the proportion of international medical graduates taking up psychiatry training (down 53.5% from 677 to 315) in the UK. This has been compensated by a 58.2% (607 to 960) increase in those who had primary qualification from the UK.

**Conclusions:**

The theme of the 31ST European Congress of Psychiatry: ‘social cohesion, a common goal of psychiatry’ blends well with what is needed in today’s psychiatry teaching and practice in the UK. With the role of culture and society well established in psychiatry, the pursuit for popularizing the profession should not be a ‘one size fits all’ approach but a more targeted approach to ensure that there is greater diversity among the available psychiatrists for patients to choose and benefit from.

**Disclosure of Interest:**

None Declared

